# Supplementary material for: Attenuation of dopamine-modulated prefrontal value signals underlies probabilistic reward learning deficits in old age
Source: eLife. 2017 Sep 5;6:e26424. doi: 10.7554/eLife.26424 (PMC5593512; doi:10.7554/eLife.26424)
Supplement: Supplementary file 1. — Coefficients in italics represent significant correlations at p<0.05. Coefficients in bold represent significant correlations at p<0.002 (adjust Bonferroni-corrected threshold). (B) Variance in number of switches as explained by the strongest RW model and winning model. When explaining the number of switches from the individual model parameters, the parameters that weighted V (υ), Crel (κ) and forgetting rate (λ), in addition to the softmax temperature parameter (β) were found to be significant predictors. Age or other model predictors did not contribute significantly. This regression model explained the number of switches better than the RW model parameters, where only the perseveration parameter b and softmax temperature parameter β were significant predictors of number of switches. (C) Young participants have a higher learning rate in the winning Rescorla-Wagner model according to non-parametric t-tests. None of the other model parameters significantly differed between groups. [file elife-26424-supp1.docx]

Table S1 A, B, C

|  |  |  |  |
| --- | --- | --- | --- |
| parameter | wins | adaptive switches | efficient choices |
| β | *0.426* | **0.620** | **0.471** |
| ω | *0.331* | **0.569** | 0.225 |
| λ | 0.111 | **0.496** | 0.121 |
| υ | -0.006 | 0.205 | 0.049 |
| κ | *-0.430* | **-***0.453* | *-0.434* |

Table S1a. Correlation coefficients between model parameters and performance. Coefficients in italics represent significant correlations at p<0.05. Coefficients in bold represent significant correlations at p<0.002 (adjust Bonferroni-corrected threshold).

|  |  |  |  |  |  |  |  |
| --- | --- | --- | --- | --- | --- | --- | --- |
| Bayesian |  | υ | κ | $\lambda$ | β | Model R^2^ | BIC |
|  | Parameter estimate | 0.831 | 0.428 | -0.208 | -0.419 | 0.90 | 451 |
|  | p | <0.001 | <0.001 | 0.013 | <0.001 |  |  |
|  |  |  |  |  |  |  |  |
|  |  | b | β |  |  | Model R^2^ | BIC |
| RW | Parameter estimate | -0.479, | -0.712 |  |  | 0.70 | 502 |
|  | p | <0.001 | <0.001 |  |  |  |  |
|  |  |  |  |  |  |  |  |
| F-statistic of the reduction in residuals | | | 47.7  p<0.001 |  |  |  |  |

Table S1b. Variance in number of switches as explained by the strongest RW model and winning model. When explaining the number of switches from the individual model parameters, the parameters that weighted V (υ), C_rel_ (κ) and forgetting rate ($\lambda$), in addition to the softmax temperature parameter (β) were found to be significant predictors. Age or other model predictors did not contribute significantly. This regression model explained the number of switches better than the RW model parameters, where only the perseveration parameter b and softmax temperature parameter β were significant predictors of number of switches.

| **parameter** | **group** | **median** | **p** |
| --- | --- | --- | --- |
| Β | old | 3.61 | 0.30 |
|  | young | 3.65 |  |
| Α | old | 0.76 | 0.04 |
|  | young | 0.86 |  |
| b | old | -0.10 | 0.35 |
|  | young | -0.12 |  |
| ϕ | old | 0.54 | 0.16 |
|  | young | 0.60 |  |

Table S1c. Young participants have a higher learning rate in the winning Rescorla-Wagner model according to non-parametric t-tests. None of the other model parameters significantly differed between groups.
