# Supplementary material for: Attenuation of dopamine-modulated prefrontal value signals underlies probabilistic reward learning deficits in old age
Source: eLife. 2017 Sep 5;6:e26424. doi: 10.7554/eLife.26424 (PMC5593512; doi:10.7554/eLife.26424)
Supplement: Supplementary file 2. — (B) Partial correlation matrix showing correlation coefficients between the binding potential in the different PET ROIs and their p-values after controlling for age. [file elife-26424-supp2.docx]

| **Table S2a** |  |  |  |  |  |  |  |
| --- | --- | --- | --- | --- | --- | --- | --- |
| **ROI** |  | Monetary gains | β | ω | λ | υ | κ |
| Nacc | correlation coefficient | 0.048 | -0.024 | -0.004 | -0.241 | -0.081 | -0.207 |
|  | p | 0.728 | 0.862 | 0.976 | 0.077 | 0.556 | 0.130 |
| Caudate | correlation coefficient | 0.042 | 0.097 | -0.033 | -0.206 | -0.194 | 0.107 |
|  | p | 0.759 | 0.479 | 0.810 | 0.132 | 0.156 | 0.436 |
| Putamen | correlation coefficient | 0.020 | 0.053 | -0.072 | -0.276 | -0.154 | -0.010 |
|  | p | 0.883 | 0.703 | 0.599 | 0.041 | 0.260 | 0.942 |
| vmPFC | correlation coefficient | 0.074 | 0.112 | -0.151 | -0.168 | 0.071 | -0.074 |
|  | p | 0.590 | 0.417 | 0.271 | 0.221 | 0.605 | 0.593 |
| dlPFC | correlation coefficient | 0.036 | 0.110 | -0.175 | -0.256 | -0.015 | -0.026 |
|  | p | 0.795 | 0.422 | 0.202 | 0.059 | 0.915 | 0.852 |
| vlPFC | correlation coefficient | 0.009 | 0.095 | -0.154 | -0.305 | -0.070 | 0.031 |
|  | p | 0.949 | 0.488 | 0.261 | 0.023 | 0.614 | 0.820 |
| OFC | correlation coefficient | 0.188 | 0.076 | -0.130 | -0.212 | 0.082 | -0.007 |
|  | p | 0.168 | 0.581 | 0.344 | 0.121 | 0.553 | 0.960 |

Table S2a. No significant correlations between model parameters and dopamine D1 receptor density in any ROI after controlling for age at Bonferroni-corrected threshold of 0.0014.

**Table S2b**

|  | Nacc | Caudate | Putamen | vmPFC | dlPFC | vlPFC | OFC |
| --- | --- | --- | --- | --- | --- | --- | --- |
| Nacc | 1.00 | 0.496 | 0.644 | 0.650 | 0.607 | 0.441 | 0.674 |
|  |  | <0.001 | <0.001 | <0.001 | <0.001 | 0.001 | <0.001 |
| Caudate |  | 1.00 | 0.891 | 0.558 | 0.655 | 0.691 | 0.434 |
|  |  |  | <0.001 | <0.001 | <0.001 | <0.001 | 0.001 |
| Putamen |  |  | 1.00 | 0.642 | 0.715 | 0.670 | 0.532 |
|  |  |  |  | <0.001 | <0.001 | <0.001 | <0.001 |
| vmPFC |  |  |  | 1.00 | 0.911 | 0.689 | 0.770 |
|  |  |  |  |  | <0.001 | <0.001 | <0.001 |
| dlPFC |  |  |  |  | 1.00 | 0.886 | 0.707 |
|  |  |  |  |  |  | <0.001 | <0.001 |
| vlPFC |  |  |  |  |  | 1.00 | 0.530 |
|  |  |  |  |  |  |  | <0.001 |
| OFC |  |  |  |  |  |  | 1.00 |

Table S2b. Partial correlation matrix showing correlation coefficients between the binding potential in the different PET ROIs and their p-values after controlling for age.
