# Supplementary material for: Attenuation of dopamine-modulated prefrontal value signals underlies probabilistic reward learning deficits in old age
Source: eLife. 2017 Sep 5;6:e26424. doi: 10.7554/eLife.26424 (PMC5593512; doi:10.7554/eLife.26424)
Supplement: Supplementary file 3. [file elife-26424-supp3.docx]

| **Table S3** |  |  |  |  |  |  |  |
| --- | --- | --- | --- | --- | --- | --- | --- |
| Q positive (choice) |  |  |  |  |  |  |  |
|  |  |  |  |  |  | cluster | peak |
|  | x | y | z | k | z-score | p(FWE-corr) | p(FWE-corr) |
| Left precuneus | -22 | -52 | 12 | 1845 | 6.14 | <0.001 | <0.001 |
| Right precuneus | 12 | -52 | 16 |  | 5.62 |  | <0.001 |
| Right hippocampus | 34 | -36 | -4 | 121 | 5.49 | <0.001 | 0.001 |
| vmPFC | -2 | 50 | -8 | 187 | 5.44 | <0.001 | 0.001 |
| Right cuneus | 12 | -80 | 26 | 82 | 5.01 | 0.001 | 0.008 |
| Q negative (choice) |  |  |  |  |  |  |  |
|  |  |  |  |  |  | cluster | peak |
|  | x | y | z | k | z-score | p(FWE-corr) | p(FWE-corr) |
| Right insula | 34 | 24 | -2 | 430 | 6.47 | <0.001 | <0.001 |
| left IPL | -50 | -44 | 46 | 918 | 6.05 | <0.001 | <0.001 |
| Left insula | -32 | 20 | 0 | 248 | 5.87 | <0.001 | <0.001 |
| Left cerebellum | -38 | -60 | -30 | 494 | 5.69 | <0.001 | <0.001 |
| right rlPFC | 32 | 50 | 10 | 1646 | 5.66 | <0.001 | <0.001 |
| right IPL | 58 | -44 | 26 | 665 | 5.54 | <0.001 | <0.001 |
| SMA | 4 | 24 | 46 | 513 | 5.50 | <0.001 | <0.001 |
| left rlPFC | -38 | 50 | 6 | 199 | 5.45 | <0.001 | 0.001 |
|  |  |  |  |  |  |  |  |

Table S3. Coordinates of clusters responsive to Q at the time of choice
